# 6-Benzyl-3-[(6-chloro­pyridin-3-yl)meth­yl]-6,7-dihydro-3*H*-1,2,3-triazolo[4,5-*d*]pyrimidin-7-imine

**DOI:** 10.1107/S1600536811047568

**Published:** 2011-11-16

**Authors:** Dong-Feng Pan, Xiao-Bao Chen, Hai-Tao Gao, Chun Feng, Ping Chen

**Affiliations:** a Department of Oncology, Renmin Hospital, Hubei University of Medicine, Shiyan 442000, Hubei, People’s Republic of China; b Institute of Medicinal Chemistry, Hubei University of Medicine, Shiyan 442000, Hubei, People’s Republic of China

## Abstract

The title compound, C_17_H_14_ClN_7_, crystallizes with two independent mol­ecules in the asymmetric unit. Inter­molecular N—H⋯N and C—H⋯N hydrogen bonds contribute to the stability of the crystal structure. In addition, weak C—H⋯π and π–π stacking [centroid–centroid distances of 3.699 (8) and 3.699 (6) Å] interactions are observed.

## Related literature

For the biological activity of 1,2,3-triazoles, see: Santana *et al.* (2002 ▶); Zhao *et al.* (2005 ▶).
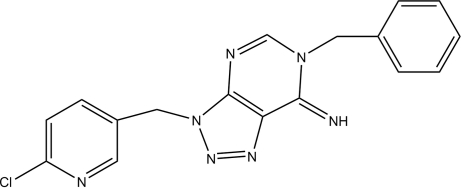


## Experimental

### 

#### Crystal data

C_17_H_14_ClN_7_
*M* *_r_* = 351.80
Triclinic, 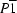

*a* = 6.1090 (7) Å
*b* = 8.9537 (11) Å
*c* = 31.292 (4) Å
α = 83.141 (1)°
β = 88.896 (1)°
γ = 75.184 (1)°
*V* = 1642.8 (3) Å^3^
*Z* = 4
Mo *K*α radiation
μ = 0.25 mm^−1^
*T* = 296 K
0.47 × 0.39 × 0.21 mm

#### Data collection

Bruker SMART APEX CCD area-detector diffractometer
Absorption correction: multi-scan (*SADABS*; Sheldrick, 2001 ▶) *T*_min_ = 0.892, *T*_max_ = 0.950
12520 measured reflections
6071 independent reflections
4578 reflections with *I* > 2σ(*I*)
*R*_int_ = 0.018

#### Refinement

*R*[*F* ^2^ > 2σ(*F* ^2^)] = 0.047
*wR*(*F* ^2^) = 0.123
*S* = 1.03
6071 reflections
451 parameters
H-atom parameters constrained
Δρ_max_ = 0.46 e Å^−3^
Δρ_min_ = −0.52 e Å^−3^

### 

Data collection: *SMART* (Bruker, 2000 ▶); cell refinement: *SAINT-Plus* (Bruker, 2000 ▶); data reduction: *SAINT-Plus*; program(s) used to solve structure: *SHELXS97* (Sheldrick, 2008 ▶); program(s) used to refine structure: *SHELXL97* (Sheldrick, 2008 ▶); molecular graphics: *SHELXTL* (Sheldrick, 2008 ▶); software used to prepare material for publication: *SHELXTL*.

## Supplementary Material

Crystal structure: contains datablock(s) global, I. DOI: 10.1107/S1600536811047568/bt5710sup1.cif

Structure factors: contains datablock(s) I. DOI: 10.1107/S1600536811047568/bt5710Isup2.hkl

Supplementary material file. DOI: 10.1107/S1600536811047568/bt5710Isup3.cml

Additional supplementary materials:  crystallographic information; 3D view; checkCIF report

## Figures and Tables

**Table 1:** Hydrogen-bond geometry (Å, °) *Cg*1 and *Cg*4 are the centroids of the N2–N4/C8/C7 and C12–C17 rings, respectively.

*D*—H⋯*A*	*D*—H	H⋯*A*	*D*⋯*A*	*D*—H⋯*A*
N14—H14*A*⋯N5^i^	0.86	2.60	3.457 (3)	173
N7—H7*A*⋯N12^ii^	0.86	2.53	3.389 (3)	174
C5—H5⋯N14^iii^	0.93	2.58	3.506 (3)	177
C10—H10⋯N11^iii^	0.93	2.42	3.240 (3)	147
C27—H27⋯N4^iv^	0.93	2.36	3.191 (3)	149
C23—H23*A*⋯*Cg*4^v^	0.97	2.58	3.509 (3)	160
C28—H28*B*⋯*Cg*1	0.97	2.93	3.506 (3)	120

Symmetry codes: (i) 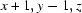; (ii) 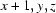; (iii) 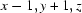; (iv) 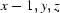; (v) 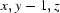.

**Fig. 1.**
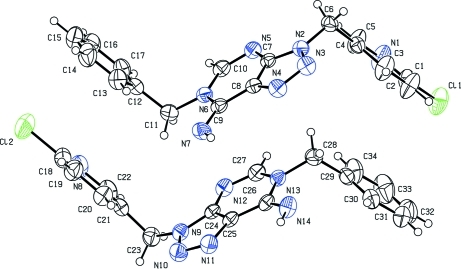
View of the molecular structure of (I), showing the atom labelling schemeand with displacement ellipsoids drawn at the 50% probability level.

**Fig. 2.**
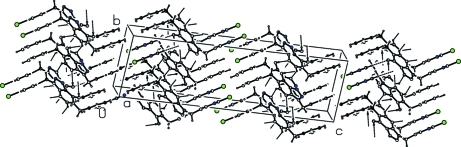
A partial view of the crystal packing of (I), showing the formation of N—H···N hydrogen-bonds as dashed lines.

## supplementary crystallographic information

### Comment

Neonicotinoids are a promising class of insecticides with excellent chemical and biological properties. 1,2,3-Triazoles have also received much attention because of their wide range of applications and biological activities such as anti-HIV and antimicrobial agents (Santana *et al.*, 2002; Zhao *et al.*, 2005). We report here the crystal structure of (I) (Fig. 1, Table 1), which was synthesized by introducing a pyridine ring into a 1,2,3-triazolo[4,5-*d*]pyrimidine molecular framework.

Significant intremolecular N—H···N and intramolecular C—H···N contribute strongly to the stability of the molecular configuration (Table 1). The crystal structure (Fig. 2) is stabilized by weak intermolecular C—H···π hydrogen bonds (Table 1) and by π— π stacking interactions with centroid-centroid separations of 3.699 (8) and 3.699 (6) Å for *Cg*3···*Cg*6^i^ and *Cg*6···*Cg*3^ii^, respectively, where *Cg*3 and *Cg*6 are the centroids of rings N5/C7—C9/N6/C10 and N9—N11/C25—C24, respectively [symmetry code: (i) *X*, 1+Y, *Z*, (ii) 3X, -1+Y, *Z*,].

### Experimental

To a solution of ethyl *N*-3-((6-chloropyridin-3-yl)methyl)-5-cyano-3*H*- 1,2,3-triazol-4-ylformimidate (2 mmol) in anhydrous acetonitrile (15 ml) was added dropwise benzylamine (2 mmol) in anhydrous acetonitrile (6 ml) at room temperature. The mixture was stirred at room temperature until the reaction finished (monitored by thin layer chromatography),the solid was filtered and recrystallized from anhydrous acetonitrile to give the title compound(yield 83%). A colourless crystal grown from anhydrous acetonitrile was selected for X-ray structure analysis.

### Refinement

H atoms were placed in calculated positions, with C—H distances in the range 0.93–0.97 Å and N—H distances of 0.86 Å, and included in the final cycles of refinement using a riding-model approximation, with *U*_iso_(H) = 1.2–1.5*U*_eq_(carrier atom). A rotating group model was used for the methyl groups.

### Crystal data

**Table:**

C_17_H_14_ClN_7_	*Z* = 4
*M**_r_* = 351.80	*F*(000) = 728
Triclinic, *P*1	*D*_x_ = 1.422 Mg m^−^^3^
*a* = 6.1090 (7) Å	Mo *K*α radiation, λ = 0.71073 Å
*b* = 8.9537 (11) Å	Cell parameters from 4552 reflections
*c* = 31.292 (4) Å	θ = 2.4–25.5°
α = 83.141 (1)°	µ = 0.25 mm^−^^1^
β = 88.896 (1)°	*T* = 296 K
γ = 75.184 (1)°	Block, colourless
*V* = 1642.8 (3) Å^3^	0.47 × 0.39 × 0.21 mm

### Data collection

**Table:**

Bruker SMART APEX CCD area-detector diffractometer	6071 independent reflections
Radiation source: fine-focus sealed tube	4578 reflections with *I* > 2σ(*I*)
graphite	*R*_int_ = 0.018
φ and ω scans	θ_max_ = 25.5°, θ_min_ = 2.4°
Absorption correction: multi-scan (*SADABS*; Sheldrick, 2001)	*h* = −7→7
*T*_min_ = 0.892, *T*_max_ = 0.950	*k* = −10→10
12520 measured reflections	*l* = −37→37

### Refinement

**Table:**

Refinement on *F*^2^	Primary atom site location: structure-invariant direct methods
Least-squares matrix: full	Secondary atom site location: difference Fourier map
*R*[*F*^2^ > 2σ(*F*^2^)] = 0.047	Hydrogen site location: inferred from neighbouring sites
*wR*(*F*^2^) = 0.123	H-atom parameters constrained
*S* = 1.03	*w* = 1/[σ^2^(*F*_o_^2^) + (0.0474*P*)^2^ + 0.785*P*] where *P* = (*F*_o_^2^ + 2*F*_c_^2^)/3
6071 reflections	(Δ/σ)_max_ < 0.001
451 parameters	Δρ_max_ = 0.46 e Å^−^^3^
0 restraints	Δρ_min_ = −0.52 e Å^−^^3^

### Special details

**Table:**

Geometry. All e.s.d.'s (except the e.s.d. in the dihedral angle between two l.s. planes)are estimated using the full covariance matrix. The cell e.s.d.'s are takeninto account individually in the estimation of e.s.d.'s in distances, anglesand torsion angles; correlations between e.s.d.'s in cell parameters are onlyused when they are defined by crystal symmetry. An approximate (isotropic)treatment of cell e.s.d.'s is used for estimating e.s.d.'s involving l.s. planes.
Refinement. Refinement of *F*^2^ against ALL reflections. The weighted *R*-factor *wR* andgoodness of fit *S* are based on *F*^2^, conventional *R*-factors *R* are basedon *F*, with *F* set to zero for negative *F*^2^. The threshold expression of*F*^2^ > σ(*F*^2^) is used only for calculating *R*-factors(gt) *etc*. and isnot relevant to the choice of reflections for refinement. *R*-factors basedon *F*^2^ are statistically about twice as large as those based on *F*, and *R*-factors based on ALL data will be even larger.

### Fractional atomic coordinates and isotropic or equivalent isotropic displacement parameters (Å^2^)

**Table:**

	*x*	*y*	*z*	*U*_iso_*/*U*_eq_	
C1	0.7744 (4)	0.6857 (3)	0.05951 (7)	0.0532 (6)	
C2	1.0038 (4)	0.6342 (4)	0.05984 (9)	0.0751 (8)	
H2	1.0789	0.5761	0.0387	0.090*	
C3	1.1206 (4)	0.6708 (3)	0.09232 (8)	0.0662 (7)	
H3	1.2777	0.6365	0.0937	0.079*	
C4	1.0060 (3)	0.7576 (2)	0.12262 (6)	0.0405 (5)	
C5	0.7759 (4)	0.8021 (3)	0.11902 (8)	0.0650 (7)	
H5	0.6959	0.8597	0.1398	0.078*	
C6	1.1269 (4)	0.8060 (2)	0.15811 (7)	0.0469 (5)	
H6A	1.2490	0.8478	0.1458	0.056*	
H6B	1.0219	0.8879	0.1713	0.056*	
C7	1.1164 (3)	0.6205 (2)	0.22548 (6)	0.0364 (4)	
C8	1.2736 (3)	0.4913 (2)	0.24336 (6)	0.0376 (4)	
C9	1.2208 (3)	0.3953 (2)	0.28042 (7)	0.0400 (5)	
C10	0.8563 (4)	0.5919 (2)	0.27273 (7)	0.0450 (5)	
H10	0.7126	0.6239	0.2842	0.054*	
C11	0.8987 (4)	0.3774 (3)	0.33020 (7)	0.0511 (5)	
H11A	0.9946	0.2727	0.3365	0.061*	
H11B	0.7495	0.3695	0.3224	0.061*	
C12	0.8811 (4)	0.4588 (2)	0.36991 (7)	0.0436 (5)	
C13	1.0709 (4)	0.4498 (3)	0.39458 (8)	0.0593 (6)	
H13	1.2116	0.3913	0.3868	0.071*	
C14	1.0532 (5)	0.5267 (4)	0.43057 (9)	0.0757 (8)	
H14	1.1818	0.5197	0.4469	0.091*	
C15	0.8475 (6)	0.6135 (4)	0.44248 (9)	0.0779 (8)	
H15	0.8365	0.6661	0.4667	0.094*	
C16	0.6579 (5)	0.6227 (3)	0.41866 (9)	0.0712 (8)	
H16	0.5180	0.6812	0.4268	0.085*	
C17	0.6731 (4)	0.5455 (3)	0.38252 (8)	0.0552 (6)	
H17	0.5433	0.5517	0.3666	0.066*	
C18	0.7452 (5)	0.1471 (3)	0.44535 (8)	0.0659 (7)	
C19	0.9630 (5)	0.0774 (3)	0.43658 (8)	0.0661 (7)	
H19	1.0823	0.0863	0.4533	0.079*	
C20	1.0010 (4)	−0.0071 (3)	0.40197 (8)	0.0562 (6)	
H20	1.1479	−0.0581	0.3952	0.067*	
C21	0.8219 (3)	−0.0163 (2)	0.37733 (7)	0.0443 (5)	
C22	0.6101 (4)	0.0635 (3)	0.38887 (8)	0.0640 (7)	
H22	0.4875	0.0619	0.3719	0.077*	
C23	0.8480 (4)	−0.1146 (3)	0.34092 (7)	0.0552 (6)	
H23A	0.8724	−0.2227	0.3529	0.066*	
H23B	0.7077	−0.0859	0.3244	0.066*	
C24	1.0343 (3)	0.0021 (2)	0.27690 (6)	0.0372 (4)	
C25	1.2463 (3)	−0.0436 (2)	0.26003 (6)	0.0384 (5)	
C26	1.3093 (3)	0.0383 (2)	0.22143 (6)	0.0403 (5)	
C27	0.9221 (4)	0.1999 (3)	0.22685 (7)	0.0471 (5)	
H27	0.8137	0.2863	0.2144	0.057*	
C28	1.1550 (4)	0.2696 (3)	0.16744 (7)	0.0534 (6)	
H28A	1.3145	0.2663	0.1644	0.064*	
H28B	1.0744	0.3756	0.1711	0.064*	
C29	1.0716 (4)	0.2268 (3)	0.12683 (7)	0.0484 (5)	
C30	1.2164 (5)	0.1361 (3)	0.10043 (8)	0.0645 (7)	
H30	1.3691	0.0994	0.1078	0.077*	
C31	1.1373 (7)	0.0985 (4)	0.06286 (9)	0.0887 (10)	
H31	1.2368	0.0369	0.0453	0.106*	
C32	0.9140 (8)	0.1520 (5)	0.05176 (11)	0.0997 (12)	
H32	0.8607	0.1261	0.0267	0.120*	
C33	0.7689 (6)	0.2435 (5)	0.07731 (11)	0.0973 (11)	
H33	0.6169	0.2808	0.0694	0.117*	
C34	0.8450 (5)	0.2812 (4)	0.11462 (9)	0.0743 (8)	
H34	0.7442	0.3437	0.1318	0.089*	
Cl1	0.61762 (13)	0.64380 (11)	0.01849 (2)	0.0860 (3)	
Cl2	0.6881 (2)	0.25203 (12)	0.48969 (3)	0.1162 (4)	
N1	0.6569 (3)	0.7684 (3)	0.08754 (7)	0.0709 (7)	
N2	1.2195 (3)	0.67680 (19)	0.19114 (5)	0.0416 (4)	
N3	1.4337 (3)	0.5854 (2)	0.18785 (6)	0.0517 (5)	
N4	1.4664 (3)	0.4726 (2)	0.21982 (6)	0.0489 (4)	
N5	0.9010 (3)	0.6798 (2)	0.23921 (6)	0.0445 (4)	
N6	0.9931 (3)	0.45918 (19)	0.29311 (5)	0.0418 (4)	
N7	1.3390 (3)	0.2699 (2)	0.30078 (6)	0.0523 (5)	
H7A	1.4758	0.2324	0.2929	0.063*	
N8	0.5685 (4)	0.1438 (3)	0.42303 (8)	0.0766 (7)	
N9	1.0329 (3)	−0.1005 (2)	0.31192 (6)	0.0447 (4)	
N10	1.2371 (3)	−0.2069 (2)	0.31669 (6)	0.0563 (5)	
N11	1.3668 (3)	−0.1714 (2)	0.28523 (6)	0.0524 (5)	
N12	0.8615 (3)	0.1264 (2)	0.26147 (6)	0.0461 (4)	
N13	1.1252 (3)	0.1660 (2)	0.20643 (5)	0.0420 (4)	
N14	1.4930 (3)	0.0109 (2)	0.20044 (6)	0.0561 (5)	
H14A	1.6030	−0.0666	0.2098	0.067*	

### Atomic displacement parameters (Å^2^)

**Table:**

	*U*^11^	*U*^22^	*U*^33^	*U*^12^	*U*^13^	*U*^23^
C1	0.0487 (13)	0.0755 (16)	0.0398 (12)	−0.0229 (12)	−0.0053 (10)	−0.0075 (11)
C2	0.0539 (15)	0.116 (2)	0.0602 (17)	−0.0143 (15)	0.0059 (12)	−0.0445 (16)
C3	0.0364 (12)	0.105 (2)	0.0589 (16)	−0.0132 (13)	0.0027 (11)	−0.0300 (15)
C4	0.0400 (11)	0.0442 (12)	0.0372 (11)	−0.0129 (9)	−0.0031 (9)	0.0012 (9)
C5	0.0449 (13)	0.096 (2)	0.0513 (15)	−0.0035 (13)	−0.0020 (11)	−0.0300 (14)
C6	0.0544 (13)	0.0421 (12)	0.0446 (12)	−0.0152 (10)	−0.0113 (10)	0.0008 (10)
C7	0.0368 (10)	0.0358 (10)	0.0361 (11)	−0.0063 (8)	−0.0076 (8)	−0.0084 (8)
C8	0.0349 (10)	0.0354 (11)	0.0413 (11)	−0.0049 (8)	−0.0075 (8)	−0.0075 (9)
C9	0.0396 (11)	0.0386 (11)	0.0424 (11)	−0.0078 (9)	−0.0074 (9)	−0.0105 (9)
C10	0.0386 (11)	0.0459 (12)	0.0482 (13)	−0.0032 (10)	−0.0027 (9)	−0.0121 (10)
C11	0.0552 (13)	0.0474 (13)	0.0540 (14)	−0.0194 (11)	0.0015 (11)	−0.0056 (10)
C12	0.0441 (12)	0.0431 (12)	0.0432 (12)	−0.0134 (9)	0.0016 (9)	0.0015 (9)
C13	0.0510 (14)	0.0684 (16)	0.0563 (15)	−0.0123 (12)	−0.0047 (11)	−0.0040 (12)
C14	0.082 (2)	0.099 (2)	0.0527 (16)	−0.0371 (18)	−0.0139 (14)	−0.0037 (15)
C15	0.108 (3)	0.087 (2)	0.0473 (16)	−0.0378 (19)	0.0141 (16)	−0.0163 (14)
C16	0.0766 (19)	0.0735 (18)	0.0590 (17)	−0.0124 (15)	0.0256 (15)	−0.0085 (14)
C17	0.0476 (13)	0.0652 (15)	0.0502 (14)	−0.0140 (11)	0.0048 (11)	0.0021 (12)
C18	0.083 (2)	0.0596 (16)	0.0455 (14)	−0.0034 (14)	0.0053 (14)	−0.0021 (12)
C19	0.0695 (17)	0.0726 (18)	0.0566 (16)	−0.0161 (14)	−0.0073 (13)	−0.0118 (13)
C20	0.0439 (12)	0.0642 (15)	0.0600 (15)	−0.0109 (11)	0.0012 (11)	−0.0114 (12)
C21	0.0418 (11)	0.0467 (12)	0.0422 (12)	−0.0116 (10)	0.0029 (9)	0.0042 (9)
C22	0.0448 (13)	0.0817 (19)	0.0572 (16)	−0.0064 (13)	−0.0023 (11)	0.0039 (14)
C23	0.0555 (14)	0.0672 (16)	0.0504 (14)	−0.0300 (12)	0.0049 (11)	−0.0068 (12)
C24	0.0353 (10)	0.0394 (11)	0.0378 (11)	−0.0076 (9)	−0.0036 (8)	−0.0124 (9)
C25	0.0338 (10)	0.0422 (11)	0.0386 (11)	−0.0040 (9)	−0.0044 (8)	−0.0141 (9)
C26	0.0356 (11)	0.0485 (12)	0.0380 (11)	−0.0082 (9)	−0.0052 (9)	−0.0148 (9)
C27	0.0425 (12)	0.0456 (12)	0.0473 (13)	0.0019 (10)	−0.0089 (10)	−0.0092 (10)
C28	0.0629 (14)	0.0491 (13)	0.0512 (14)	−0.0205 (11)	−0.0051 (11)	−0.0036 (11)
C29	0.0584 (14)	0.0481 (13)	0.0411 (12)	−0.0216 (11)	−0.0037 (10)	0.0036 (10)
C30	0.0801 (18)	0.0614 (16)	0.0479 (14)	−0.0142 (14)	0.0008 (13)	0.0014 (12)
C31	0.143 (3)	0.075 (2)	0.0474 (17)	−0.026 (2)	0.0014 (19)	−0.0092 (14)
C32	0.155 (4)	0.106 (3)	0.0534 (19)	−0.065 (3)	−0.030 (2)	0.0045 (18)
C33	0.090 (2)	0.143 (3)	0.068 (2)	−0.055 (2)	−0.0297 (19)	0.014 (2)
C34	0.0637 (17)	0.103 (2)	0.0550 (16)	−0.0236 (16)	−0.0086 (13)	0.0022 (15)
Cl1	0.0792 (5)	0.1341 (7)	0.0573 (4)	−0.0421 (5)	−0.0164 (3)	−0.0258 (4)
Cl2	0.1700 (10)	0.1012 (7)	0.0660 (5)	−0.0061 (6)	0.0226 (6)	−0.0308 (5)
N1	0.0398 (11)	0.1159 (19)	0.0575 (13)	−0.0116 (11)	−0.0059 (10)	−0.0288 (13)
N2	0.0397 (9)	0.0416 (10)	0.0429 (10)	−0.0091 (8)	−0.0082 (8)	−0.0044 (8)
N3	0.0401 (10)	0.0565 (12)	0.0560 (12)	−0.0094 (9)	−0.0023 (8)	−0.0030 (10)
N4	0.0371 (10)	0.0501 (11)	0.0552 (12)	−0.0043 (8)	−0.0028 (8)	−0.0034 (9)
N5	0.0398 (10)	0.0439 (10)	0.0448 (10)	−0.0007 (8)	−0.0054 (8)	−0.0060 (8)
N6	0.0426 (10)	0.0393 (9)	0.0432 (10)	−0.0086 (8)	−0.0015 (8)	−0.0068 (8)
N7	0.0487 (11)	0.0425 (11)	0.0587 (12)	−0.0008 (9)	−0.0110 (9)	0.0006 (9)
N8	0.0662 (15)	0.0866 (17)	0.0609 (15)	0.0077 (12)	0.0152 (12)	−0.0074 (12)
N9	0.0426 (10)	0.0483 (11)	0.0435 (10)	−0.0101 (8)	−0.0012 (8)	−0.0091 (8)
N10	0.0552 (12)	0.0518 (12)	0.0534 (12)	−0.0003 (9)	−0.0033 (10)	−0.0013 (9)
N11	0.0447 (10)	0.0541 (12)	0.0491 (11)	0.0040 (9)	−0.0029 (9)	−0.0052 (9)
N12	0.0349 (9)	0.0519 (11)	0.0477 (11)	−0.0017 (8)	−0.0030 (8)	−0.0106 (9)
N13	0.0424 (10)	0.0438 (10)	0.0391 (10)	−0.0080 (8)	−0.0043 (8)	−0.0077 (8)
N14	0.0400 (10)	0.0775 (14)	0.0489 (11)	−0.0091 (10)	0.0025 (9)	−0.0138 (10)

### Geometric parameters (Å, °)

**Table:**

C1—N1	1.301 (3)	C19—C20	1.377 (3)
C1—C2	1.359 (3)	C19—H19	0.9300
C1—Cl1	1.747 (2)	C20—C21	1.376 (3)
C2—C3	1.370 (3)	C20—H20	0.9300
C2—H2	0.9300	C21—C22	1.373 (3)
C3—C4	1.366 (3)	C21—C23	1.503 (3)
C3—H3	0.9300	C22—N8	1.343 (3)
C4—C5	1.363 (3)	C22—H22	0.9300
C4—C6	1.509 (3)	C23—N9	1.457 (3)
C5—N1	1.342 (3)	C23—H23A	0.9700
C5—H5	0.9300	C23—H23B	0.9700
C6—N2	1.459 (3)	C24—N9	1.345 (3)
C6—H6A	0.9700	C24—C25	1.370 (3)
C6—H6B	0.9700	C24—N12	1.370 (3)
C7—N2	1.346 (3)	C25—N11	1.360 (3)
C7—C8	1.368 (3)	C25—C26	1.436 (3)
C7—N5	1.369 (2)	C26—N14	1.273 (3)
C8—N4	1.361 (3)	C26—N13	1.423 (3)
C8—C9	1.441 (3)	C27—N12	1.297 (3)
C9—N7	1.273 (3)	C27—N13	1.365 (3)
C9—N6	1.431 (3)	C27—H27	0.9300
C10—N5	1.302 (3)	C28—N13	1.480 (3)
C10—N6	1.359 (3)	C28—C29	1.504 (3)
C10—H10	0.9300	C28—H28A	0.9700
C11—N6	1.484 (3)	C28—H28B	0.9700
C11—C12	1.503 (3)	C29—C30	1.375 (3)
C11—H11A	0.9700	C29—C34	1.389 (3)
C11—H11B	0.9700	C30—C31	1.389 (4)
C12—C13	1.386 (3)	C30—H30	0.9300
C12—C17	1.386 (3)	C31—C32	1.362 (5)
C13—C14	1.377 (4)	C31—H31	0.9300
C13—H13	0.9300	C32—C33	1.362 (5)
C14—C15	1.369 (4)	C32—H32	0.9300
C14—H14	0.9300	C33—C34	1.375 (4)
C15—C16	1.368 (4)	C33—H33	0.9300
C15—H15	0.9300	C34—H34	0.9300
C16—C17	1.384 (4)	N2—N3	1.364 (2)
C16—H16	0.9300	N3—N4	1.313 (3)
C17—H17	0.9300	N7—H7A	0.8600
C18—N8	1.306 (4)	N9—N10	1.361 (2)
C18—C19	1.356 (4)	N10—N11	1.312 (3)
C18—Cl2	1.747 (3)	N14—H14A	0.8600
			
N1—C1—C2	125.0 (2)	C20—C21—C23	123.2 (2)
N1—C1—Cl1	115.55 (17)	N8—C22—C21	124.3 (2)
C2—C1—Cl1	119.48 (19)	N8—C22—H22	117.9
C1—C2—C3	117.7 (2)	C21—C22—H22	117.9
C1—C2—H2	121.2	N9—C23—C21	114.42 (18)
C3—C2—H2	121.2	N9—C23—H23A	108.7
C4—C3—C2	119.9 (2)	C21—C23—H23A	108.7
C4—C3—H3	120.0	N9—C23—H23B	108.7
C2—C3—H3	120.0	C21—C23—H23B	108.7
C5—C4—C3	117.1 (2)	H23A—C23—H23B	107.6
C5—C4—C6	120.9 (2)	N9—C24—C25	105.25 (17)
C3—C4—C6	122.01 (19)	N9—C24—N12	127.46 (18)
N1—C5—C4	124.4 (2)	C25—C24—N12	127.29 (19)
N1—C5—H5	117.8	N11—C25—C24	108.66 (19)
C4—C5—H5	117.8	N11—C25—C26	130.20 (18)
N2—C6—C4	112.29 (17)	C24—C25—C26	121.13 (18)
N2—C6—H6A	109.1	N14—C26—N13	120.1 (2)
C4—C6—H6A	109.1	N14—C26—C25	130.2 (2)
N2—C6—H6B	109.1	N13—C26—C25	109.74 (17)
C4—C6—H6B	109.1	N12—C27—N13	128.0 (2)
H6A—C6—H6B	107.9	N12—C27—H27	116.0
N2—C7—C8	104.89 (17)	N13—C27—H27	116.0
N2—C7—N5	127.29 (18)	N13—C28—C29	113.43 (18)
C8—C7—N5	127.81 (19)	N13—C28—H28A	108.9
N4—C8—C7	109.19 (19)	C29—C28—H28A	108.9
N4—C8—C9	129.66 (18)	N13—C28—H28B	108.9
C7—C8—C9	121.10 (18)	C29—C28—H28B	108.9
N7—C9—N6	120.0 (2)	H28A—C28—H28B	107.7
N7—C9—C8	130.7 (2)	C30—C29—C34	118.2 (2)
N6—C9—C8	109.28 (17)	C30—C29—C28	121.4 (2)
N5—C10—N6	128.2 (2)	C34—C29—C28	120.4 (2)
N5—C10—H10	115.9	C29—C30—C31	120.8 (3)
N6—C10—H10	115.9	C29—C30—H30	119.6
N6—C11—C12	112.68 (17)	C31—C30—H30	119.6
N6—C11—H11A	109.1	C32—C31—C30	120.0 (3)
C12—C11—H11A	109.1	C32—C31—H31	120.0
N6—C11—H11B	109.1	C30—C31—H31	120.0
C12—C11—H11B	109.1	C31—C32—C33	120.0 (3)
H11A—C11—H11B	107.8	C31—C32—H32	120.0
C13—C12—C17	118.5 (2)	C33—C32—H32	120.0
C13—C12—C11	121.1 (2)	C32—C33—C34	120.6 (3)
C17—C12—C11	120.4 (2)	C32—C33—H33	119.7
C14—C13—C12	120.6 (2)	C34—C33—H33	119.7
C14—C13—H13	119.7	C33—C34—C29	120.5 (3)
C12—C13—H13	119.7	C33—C34—H34	119.8
C15—C14—C13	120.5 (3)	C29—C34—H34	119.8
C15—C14—H14	119.8	C1—N1—C5	116.0 (2)
C13—C14—H14	119.8	C7—N2—N3	110.05 (16)
C16—C15—C14	119.7 (3)	C7—N2—C6	129.13 (17)
C16—C15—H15	120.1	N3—N2—C6	120.61 (18)
C14—C15—H15	120.1	N4—N3—N2	107.70 (17)
C15—C16—C17	120.5 (3)	N3—N4—C8	108.16 (16)
C15—C16—H16	119.8	C10—N5—C7	110.24 (17)
C17—C16—H16	119.8	C10—N6—C9	123.29 (18)
C16—C17—C12	120.2 (2)	C10—N6—C11	117.72 (18)
C16—C17—H17	119.9	C9—N6—C11	118.99 (17)
C12—C17—H17	119.9	C9—N7—H7A	119.3
N8—C18—C19	125.3 (2)	C18—N8—C22	116.1 (2)
N8—C18—Cl2	115.6 (2)	C24—N9—N10	109.90 (17)
C19—C18—Cl2	119.1 (2)	C24—N9—C23	129.50 (19)
C18—C19—C20	117.4 (3)	N10—N9—C23	120.47 (19)
C18—C19—H19	121.3	N11—N10—N9	107.71 (17)
C20—C19—H19	121.3	N10—N11—C25	108.47 (17)
C21—C20—C19	120.1 (2)	C27—N12—C24	110.74 (18)
C21—C20—H20	119.9	C27—N13—C26	123.09 (18)
C19—C20—H20	119.9	C27—N13—C28	117.92 (18)
C22—C21—C20	116.6 (2)	C26—N13—C28	118.98 (18)
C22—C21—C23	120.0 (2)	C26—N14—H14A	119.2
			
N1—C1—C2—C3	0.6 (5)	C30—C29—C34—C33	0.6 (4)
Cl1—C1—C2—C3	179.4 (2)	C28—C29—C34—C33	179.4 (3)
C1—C2—C3—C4	−0.7 (5)	C2—C1—N1—C5	−0.7 (4)
C2—C3—C4—C5	0.9 (4)	Cl1—C1—N1—C5	−179.6 (2)
C2—C3—C4—C6	−177.7 (3)	C4—C5—N1—C1	1.0 (4)
C3—C4—C5—N1	−1.1 (4)	C8—C7—N2—N3	0.0 (2)
C6—C4—C5—N1	177.6 (2)	N5—C7—N2—N3	−179.75 (18)
C5—C4—C6—N2	107.6 (3)	C8—C7—N2—C6	174.65 (18)
C3—C4—C6—N2	−73.8 (3)	N5—C7—N2—C6	−5.1 (3)
N2—C7—C8—N4	0.2 (2)	C4—C6—N2—C7	−84.0 (3)
N5—C7—C8—N4	179.92 (18)	C4—C6—N2—N3	90.1 (2)
N2—C7—C8—C9	−177.67 (17)	C7—N2—N3—N4	−0.2 (2)
N5—C7—C8—C9	2.1 (3)	C6—N2—N3—N4	−175.36 (17)
N4—C8—C9—N7	0.4 (4)	N2—N3—N4—C8	0.3 (2)
C7—C8—C9—N7	177.7 (2)	C7—C8—N4—N3	−0.3 (2)
N4—C8—C9—N6	−177.81 (19)	C9—C8—N4—N3	177.32 (19)
C7—C8—C9—N6	−0.5 (2)	N6—C10—N5—C7	0.0 (3)
N6—C11—C12—C13	−76.1 (3)	N2—C7—N5—C10	177.90 (19)
N6—C11—C12—C17	103.6 (2)	C8—C7—N5—C10	−1.8 (3)
C17—C12—C13—C14	−0.7 (4)	N5—C10—N6—C9	1.5 (3)
C11—C12—C13—C14	179.1 (2)	N5—C10—N6—C11	−178.2 (2)
C12—C13—C14—C15	−0.1 (4)	N7—C9—N6—C10	−179.51 (19)
C13—C14—C15—C16	0.6 (4)	C8—C9—N6—C10	−1.1 (2)
C14—C15—C16—C17	−0.3 (4)	N7—C9—N6—C11	0.2 (3)
C15—C16—C17—C12	−0.5 (4)	C8—C9—N6—C11	178.59 (16)
C13—C12—C17—C16	0.9 (3)	C12—C11—N6—C10	−74.6 (2)
C11—C12—C17—C16	−178.8 (2)	C12—C11—N6—C9	105.7 (2)
N8—C18—C19—C20	−1.8 (4)	C19—C18—N8—C22	0.3 (4)
Cl2—C18—C19—C20	178.6 (2)	Cl2—C18—N8—C22	−180.0 (2)
C18—C19—C20—C21	1.1 (4)	C21—C22—N8—C18	1.9 (4)
C19—C20—C21—C22	0.8 (4)	C25—C24—N9—N10	−0.1 (2)
C19—C20—C21—C23	−175.9 (2)	N12—C24—N9—N10	−179.77 (19)
C20—C21—C22—N8	−2.4 (4)	C25—C24—N9—C23	−175.82 (19)
C23—C21—C22—N8	174.4 (2)	N12—C24—N9—C23	4.5 (3)
C22—C21—C23—N9	137.4 (2)	C21—C23—N9—C24	−87.3 (3)
C20—C21—C23—N9	−46.0 (3)	C21—C23—N9—N10	97.4 (2)
N9—C24—C25—N11	−0.2 (2)	C24—N9—N10—N11	0.4 (2)
N12—C24—C25—N11	179.42 (19)	C23—N9—N10—N11	176.60 (18)
N9—C24—C25—C26	178.91 (17)	N9—N10—N11—C25	−0.6 (2)
N12—C24—C25—C26	−1.4 (3)	C24—C25—N11—N10	0.5 (2)
N11—C25—C26—N14	1.3 (4)	C26—C25—N11—N10	−178.5 (2)
C24—C25—C26—N14	−177.6 (2)	N13—C27—N12—C24	0.2 (3)
N11—C25—C26—N13	179.98 (19)	N9—C24—N12—C27	−179.68 (19)
C24—C25—C26—N13	1.0 (2)	C25—C24—N12—C27	0.7 (3)
N13—C28—C29—C30	−96.1 (3)	N12—C27—N13—C26	−0.4 (3)
N13—C28—C29—C34	85.2 (3)	N12—C27—N13—C28	−179.0 (2)
C34—C29—C30—C31	−0.7 (4)	N14—C26—N13—C27	178.57 (19)
C28—C29—C30—C31	−179.5 (2)	C25—C26—N13—C27	−0.2 (3)
C29—C30—C31—C32	0.1 (4)	N14—C26—N13—C28	−2.8 (3)
C30—C31—C32—C33	0.6 (5)	C25—C26—N13—C28	178.38 (16)
C31—C32—C33—C34	−0.7 (5)	C29—C28—N13—C27	−86.0 (2)
C32—C33—C34—C29	0.1 (5)	C29—C28—N13—C26	95.4 (2)

### Hydrogen-bond geometry (Å, °)

**Table:**

Cg1 and Cg4 are the centroids of the N2–N4/C8/C7 and C12–C17 rings, respectively.

**Table:**

*D*—H···*A*	*D*—H	H···*A*	*D*···*A*	*D*—H···*A*
N14—H14A···N5^i^	0.86	2.60	3.457 (3)	173.
N7—H7A···N12^ii^	0.86	2.53	3.389 (3)	174.
C5—H5···N14^iii^	0.93	2.58	3.506 (3)	177
C10—H10···N11^iii^	0.93	2.42	3.240 (3)	147
C27—H27···N4^iv^	0.93	2.36	3.191 (3)	149
C23—H23A···Cg4^v^	0.97	2.58	3.509 (3)	160
C28—H28B···Cg1	0.97	2.93	3.506 (3)	120

Symmetry codes: (i) *x*+1, *y*−1, *z*; (ii) *x*+1, *y*, *z*; (iii) *x*−1, *y*+1, *z*; (iv) *x*−1, *y*, *z*; (v) *x*, *y*−1, *z*.
